# COVID-19 FAQs in paediatric and congenital cardiology: AEPC position paper

**DOI:** 10.1017/S1047951120005028

**Published:** 2021-01-07

**Authors:** Skaiste Sendzikaite, Ruth Heying, Ornella Milanesi, Katarina Hanseus, Ina Michel-Behnke

**Affiliations:** 1 Clinic of Paediatrics, Institute of Clinical Medicine, Vilnius University, Vilnius, Lithuania; 2 Department of Paediatric and Congenital Cardiology, University Hospitals Leuven, Leuven, Belgium; 3 Paediatric Cardiology Unit, Department of Child and Woman’s Health, University of Padua, Padua, Italy; 4 Department of Paediatric Cardiology, Skåne University Hospital, Lund, Sweden; 5 Department of Clinical Sciences, Lund University, Lund, Sweden; 6 Department of Paediatric Cardiology, University Hospital of Children and Adolescent Medicine, Department of Paediatric Cardiology/Paediatric Heart Center, Medical University of Vienna, Vienna, Austria

**Keywords:** COVID-19, SARS-Cov-2, Kawasaki, multisystem inflammatory syndrome, AEPC, congenital heart disease

## Abstract

The COVID-19 pandemic has had a huge influence in almost all areas of life, affecting societies, economics, and health care systems worldwide. The paediatric cardiology community is no exception. As the challenging battle with COVID-19 continues, professionals from the Association for the European Paediatric and Congenital Cardiology receive many questions regarding COVID-19 in a Paediatric and Congenital Cardiology setting. The aim of this paper is to present the AEPC position on frequently asked questions based on the most recent scientific data, as well as to frame a discussion on how to take care of our patients during this unprecedented crisis. As the times are changing quickly and information regarding COVID-19 is very dynamic, continuous collection of evidence will help guide constructive decision-making.

It is now more than a half year since the COVID-19 pandemic started to influence almost all areas of life, affecting societies, economics, and healthcare worldwide and it is likely that we will have to endure this for a long time ahead.

Many questions have arisen regarding patients with CHD, with respect to increased susceptibility to the coronavirus, the risk of more severe disease in the presence of CHD, the value of testing, and regarding recent reports on the inflammatory disease with some Kawasaki features.

The aim of this paper is to present the AEPC position on frequently asked questions regarding COVID-19 in a Paediatric and Congenital Cardiology setting, based on continuous monitoring and evaluation of scientific papers. It should be kept in mind that times are changing fast and this paper has been written with the best evidence available at the time of writing. The AEPC position will be updated in the future according to further evidence in the literature.

*Methods*: This manuscript has been reviewed and approved by the AEPC Council for publication in Cardiology in the Young as an official position paper of AEPC.

## What is known about clinical manifestation of COVID-19 in paediatric CHD patients?

According to the current state of knowledge, COVID-19 infections in children are mostly mild and self-limiting. Children are at very low risk of being admitted to the ICU. However, children under the age of 1 year seem to have a higher risk for a more severe course of the infection. Whether children with underlying cardiac diseases are more susceptible to cardiac injury remains unknown. Three percent of patients from the United States of America cohort of 177 patients infected with SARS-Cov-2 had pre-existing cardiac disease and more frequent hospital admission compared with other underlying conditions.^1^ Nevertheless, and although not scientifically proven, most children and adults with CHD presenting with severe cyanosis, pulmonary hypertension, or heart failure should be monitored more closely during the SARS-Cov-2 pandemic.

As in adults, underlying cardiac diseases with a decreased cardiac reserve and enhanced metabolic demand contribute to increased severity of respiratory symptoms and worse outcome.^2,3^ Some risk factors in the adult population for higher susceptibility to COVID-19 outcome are partly rebutted, like arterial hypertension. Deeper insights from the studies on the risk factors for mortality of COVID-19, did not prove evidence that hypertension contributes to poor outcome, even if prevalence is high.^4^

A comprehensive guidance document has been established that addresses all management aspects of cardiovascular diseases in adults during the COVID-19 pandemic.^5^

Currently, there is only scarce data on COVID-19 in children with CHD, so that, no sound data is available for a detailed risk stratification.^6–8^

## Which paediatric and grown-up CHD groups are at major risk for COVID-19 infection?

Despite the large number of cases with COVID-19, little is known about the risk and effects in children as well as adults with CHD. However, it seems that children have a substantially better prognosis than adults with cardiovascular risk factors.

According to a recent multicentre cross-sectional study from 46 centres within The United States of America and Canada, the overall ICU mortality of COVID-19 in children is less than 5%, compared with published mortalities of 50–62% in adults admitted to the ICU. These conclusions were drawn from 48 critically ill children with COVID-19, of whom more than 80% had significant comorbidities including CHD.^9^ Data from China suggests the same tendency towards better survival and outcomes from critical illness in infants and children than reported for adult patients.^10,11^

The British Congenital Cardiac Association has published recommendations for precautions for some groups with other infections in CHD patients, based on existing knowledge from diseases, which have been extrapolated to the COVID-19 situation. According to the British Congenital Cardiac Association recommendations, the following CHD patient groups are estimated to be at high risk for a severe disease course with an increased risk for a negative outcome and should be particularly strict in following the social distancing and hygiene measures:

• Single-ventricle patients.
• Infants under 1 year with unrepaired CHD requiring surgery or catheter intervention.
• Patients with chronic cyanosis (oxygen saturations <85% persistently).
• Patients with severe cardiomyopathies.
• Patients with CHD on medication for heart failure.
• Patients with pulmonary hypertension.
• Patients after heart transplantation.
• Patients with CHD and significant comorbidities, e.g., chronic kidney disease, chronic lung disease.^12^
• Grown-up CHD patients with Down’s syndrome have a 4fold increased risk to be admitted to hospital and a 10-fold increased risk for COVID-19-related death.^13^

## What is known about a potential cardiac pathology related to COVID-19 in children?

To date, cardiac pathology in children has been described in very few cases beyond the systemic inflammatory disease, and in general cases, fatality is low. Tissue damage to lungs, heart, and other organs caused by SARS-CoV-2 is induced by binding of the virus with its surface spike glycoprotein to the host angiotensin-converting enzyme 2 receptor. In the human heart, the receptors are localised at the pericytes and their injury can lead to secondary capillary endothelial dysfunction, which is responsible for cardiac pathology.^14^ Amongst several theories of protective mechanisms in the paediatric age group, the lower number and immaturity of angiotensin-converting enzyme 2 in children has been speculated to be one of the reasons for the milder nature of COVID-19.^15^

Clinical symptoms of myocardial damage in adults affected with COVID-19 are rhythm disorders, hypotension, and cardiogenic shock. Biomarkers like troponin, creatine kinase, and lactate dehydrogenase are elevated. Acute myocarditis has been suspected in adults as myocardial interstitial oedema and diffuse late gadolinium enhancement could be documented.^16^ It remains obscure whether all cardiac pathologies are related to the primary viral attack on cardiomyocytes or whether it is the result of proinflammatory cytokines and systemic inflammatory response.

Fulminant acute myocardial necrosis and mononuclear cell infiltration have been described, but only rarely have virus particles been demonstrated in myocardial tissue.^17^ Together with a timely delayed presentation of symptoms (10–15 days), these findings might be interpreted in favour of inflammation-mediated myocardial injury rather than induced by the cytopathic effect of the virus.^18^

Hyperinflammation, imbalance of renin–angiotensin–aldosterone system, and a particular form of vasculopathy, thrombotic microangiopathy are reported to contribute to increased severity of COVID-19, not only in respiratory but also in cardiac failure.^19^

In this context, impaired cardiac function, vasculitis, and septic shock as well as features of a Kawasaki disease shock syndrome have also been described in children, with distinct differences in inflammatory markers. While coronary involvement was variable, depressed left ventricular function, pericardial effusion, and arrhythmia were the main findings.^20,21^ The special characteristics of the delayed immune response after COVID-19, named MIS-C are provided in question 4.

It was recently published that SARS-CoV-2 could be isolated from cardiac tissue in a child that died within a COVID-19-related multisystem inflammatory syndrome.^22^ The timeline of the symptoms, in that case, does not rule out completely that the patient was in the acute phase of COVID-19 rather than MIS-C when myocardial evidence of the virus was detected.

## What are the possible inflammatory heart diseases related to COVID-19 in the paediatric age group?

In the frame of the actual COVID-19 pandemic, an increasing number of children were admitted for clinical symptoms of the condition termed multisystem inflammatory syndrome (also referred to as paediatric multisystem inflammatory syndrome, paediatric inflammatory multisystem syndrome temporally associated with SARS-CoV-2, paediatric hyperinflammatory syndrome, or paediatric hyperinflammatory shock).^23^ These reports came specifically from countries with a high number of SARS-CoV-2 infections, such as Italy, Spain, the United Kingdom, and the United States of America. Understanding of COVID-19 infection and multisystem inflammatory syndrome is evolving. Guidance has been issued and consistently renewed by the World Health Organization and the Centers for Disease Control and Prevention.^24,25^ An association to a SARS-CoV-2 infection or a secondary reaction has been discussed and according to the most recent literature, multisystem inflammatory syndrome manifests temporarily in association with SARS-CoV-2, appearing about 3–4 weeks after the acute infection.^26,27^ This may explain why many children had positive antibodies to SARS-CoV-2, but a negative reverse transcription-polymerase chain reaction on swab analysis at the time of multisystem inflammatory syndrome evaluation. Severe manifestation of COVID-19 in the paediatric age group is, in general, rare. The described severe cases do not present the first line with pulmonary symptoms, but with an untypical inflammatory reaction and gastrointestinal complaints. The cardiovascular system is affected by a vasculitis and in some cases by myocardial dysfunction. The clinical presentation shows overlapping features of a toxic shock syndrome and Kawasaki syndrome (or atypical one), and the criteria for Kawasaki disease do not apply fully for diagnosis.

Clinical data on multisystem inflammatory syndrome provide evidence that although multisystem inflammatory syndrome and Kawasaki disease seem to be based on an increased inflammatory reaction, multisystem inflammatory syndrome differs from other paediatric inflammatory syndromes.^23^ Some patients are described who have presented with a refractory vasodilatory shock syndrome with normal cardiac function. Not uncommonly children can present with septic or cardiogenic shock and impaired cardiac function (mainly left ventricular).

Different organisations, such as the World Health Organization, the Centers for Disease Control and Prevention, and the Royal College of Paediatrics and Child Health have published definitions of the multisystem inflammatory syndrome, which are in slight variance from each other.^20,21,28^

As a reference, Table 1 summarises the definitions for MIS-C of the WHO and CDC.

**Table 1.** WHO and CDC definitions of the multisystem inflammatory syndrome in children.

MIS-C definition of WHO	MIS-C definition of CDC
Required: all six criteria	Required: all four criteria
1. Children’s age 0–19 years.	1. Age <21 years.
2. Fever for ≥3 days.	2. Fever: documented >38.0 °C ≥ 24 hours or reported subjective fever lasting ≥24 hours.
3. Clinical signs of multisystem involvement (at least two of the following):	3. Clinical presentation consistent with MIS-C, including all of the following:
Rash or bilateral non-purulent conjunctivitis, or mucocutaneous inflammation signs (oral, hands, or feet).	Evidence of clinically severe illness requiring hospitalisation, with multisystem (>2) organ involvement.
Hypotension or shock.	Cardiovascular, renal, neurologic, haematologic, gastrointestinal, or dermatologic system.
Features of myocardial dysfunction, pericarditis, valvulitis, or coronary abnormalities (including ECHO findings or elevated troponin/NT-proBNP).	4. Laboratory incidence of inflammation including, but not limited to any of the following:
Evidence of coagulopathy (prolonged prothrombin time or partial thromboplastin time, elevated D-dimer).	Elevated: C-reactive protein, erythrocyte sedimentation rate, fibrinogen, procalcitonin, D-dimer, ferritin, lactic acid dehydrogenase, interleukin 6, neutrophils.
Acute gastrointestinal symptoms (diarrhoea, vomiting, or abdominal pain).	Reduced lymphocytes, low albumin.
4. Elevated markers of inflammation such as C-reactive protein, erythrocyte sedimentation rate, or procalcitonin.	5. Positive for current or recent SARS-CoV-2 infection by RT-PCR, serology, or antigen test; or exposure to a suspected or confirmed COVID-19 case within the 4 weeks prior to the onset of symptoms.
5. No other obvious microbial cause of inflammation, including bacterial sepsis and staphylococcal/streptococcal toxic shock syndromes.	6. No alternative plausible diagnoses.
6. Evidence of COVID-19 (RT-PCR, antigen test, or serology positive), or likely contact with patients with COVID-19.	

The definitions of multisystem inflammatory syndrome in children from the World Health Organization^25^ and Centers for Disease Control and Prevention^24^ are summarised in this table. The CDC also comments that some individuals may fulfil full or partial criteria for Kawasaki disease, but should be reported if they meet the case definition for MIS-C. MIS-C should be considered in any paediatric death with evidence of SARS-CoV-2 infection.

It seems that important differences occur in clinical and laboratory findings, which distinguish multisystem inflammatory syndrome from other paediatric inflammatory syndromes, such as Kawasaki disease, Kawasaki disease shock syndrome, and toxic shock syndrome.^23^

The following major laboratory findings are described to characterise a potential multisystem inflammatory syndrome in comparison to Kawasaki disease^29^:

• anaemia, lymphopenia,
• elevated leucocytes and neutrophils,
• normal or low platelets,
• elevated fibrinogen and ferritin,
• elevated troponin, N-terminal pro-b-type natriuretic peptide,
• decreased albumin,
• elevated interleukin 6, C-reactive protein,
• lymphocytic myocarditis.

**Figure 1.**
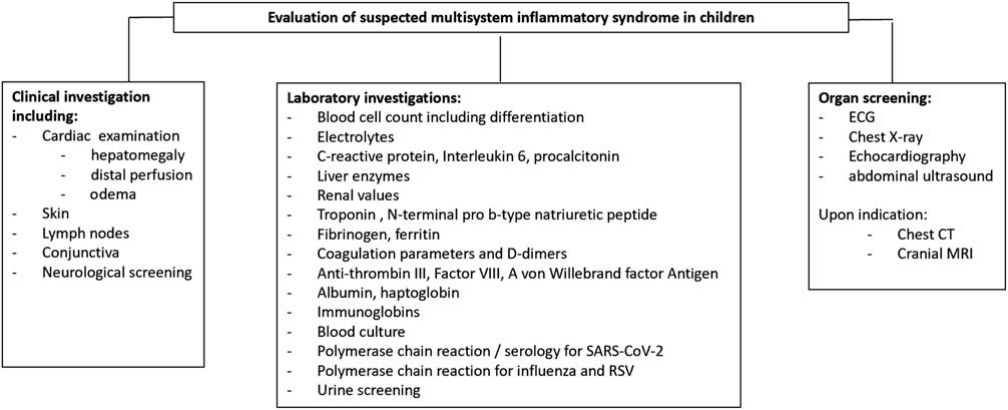
Summarises the indicated diagnostic investigations and the potentially considered ones depending on the clinical presentation in patients with suspicion of the multisystem inflammatory syndrome.

Patients with the suspected multisystem inflammatory syndrome should undergo recommended diagnostic blood, urine, virology, bacteriology, serology, echocardiography, and sonography tests at admission.^28^ Figure 1 shows a diagnostic algorithm.

Treatment strategies of the multisystem inflammatory syndrome are as follows:

- Anti-inflammatory treatment: steroids, intravenous immunoglobulin, aspirin, and possibly interleukin 6 inhibitors.
- Shock: inotropes, management of cardiac dysfunction.
- Anti-platelet and anti-coagulation therapy: enoxaparin and others.

The aetiology is not yet conclusive and might be in accordance with the inflammatory reaction seen with a delay in adults. Similar to the results of genetic analysis in Kawasaki research, the origin of the inflammation might be associated with genes that regulate the rate of production of T-cells or antibody responses or the clearing of antibody and immune complexes.^20,21,28^

## What is known about the potential management of arrhythmia syndromes and anti-arrhythmic medications in children?

SARS-CoV-2 infection may occur in patients with channelopathies, e.g., congenital long QT syndrome, Brugada syndrome, catecholaminergic polymorphic ventricular tachycardia, and short QT syndrome, with a risk of pro-arrhythmia.^5,30^

In the setting of long QT syndrome with COVID-19, the QT interval should be monitored closely because the combination of drugs (Hydroxychloroquine and Azithromycin) and stress factors (electrolyte disturbances and kidney dysfunction) may further prolong QT.^5^

Fever-triggered malignant ventricular arrhythmia is the major concern in Brugada syndrome with COVID-19, therefore, fever should be aggressively treated with paracetamol.^31^

In patients with catecholaminergic polymorphic ventricular tachycardia and COVID-19, beta-blockers and flecainide should be continued with monitoring of drug interactions with the above-mentioned drugs.^5^

## Is there an increased patient risk for cardiac surgery/cardiac intervention/heart transplantation during the COVID-19 pandemic?

The incidence of COVID-19 remains low in children. Numbers are quoted to be between 2 and 12% (depending on testing), with a generally milder course, but with variable symptoms that may mask the viral infection and cause a delay in making the diagnosis.

Despite common-sense judgement that pre-existing cardiac diseases should impose children with CHD to a higher risk, this has only rarely been demonstrated to date.^32^

The following strategies should minimise the risk of cardiac surgery during the COVID-19 pandemic:

- Prioritisation of cardiac surgery, leaving the surgical activity to urgent and emergency procedures.
- Avoid the delay of urgent cardiac surgery.
- CHD patients planned for cardiac surgery if not an emergency should have tested negative for SARS-Cov-2 reverse transcription-polymerase chain reaction.
- Test- and symptom-based precautions to avoid transmission: separation of positive-tested COVID-19 patients from non-COVID-19 patients irrespective of symptoms. Staff working in rotations.
- Full personal protective equipment should be mandatory for staff who is performing a cardiac intervention for COVID-19-positive patients.
- In CHD, patients positive for SARS-CoV-2 (test or symptoms) surgery should be delayed until the test is negative or symptoms relieved (usually 14 days) if clinically justifiable.
- Provide extensive logistics including anaesthetic and intensive care considerations to protect patients and medical staff.
- Continue transmission precautions at least 14 days after discharge from the hospital.

Paediatric heart catheterisation and intervention in COVID-19 patients have been required in exceptional circumstances. Elective cases have, in general, been postponed from the beginning of the pandemic. Meanwhile, as the numbers of affected children remain low, it seems reasonable to activate the programmes while continuing to use personal protective equipment and following institutional flow algorithms for COVID-19 patients, including switch to a negative pressure environment in the catheter laboratory if possible. A comprehensive guidance paper addresses most aspects of decision-making and resource allocation.^33^ The subgroup of adults with CHD are likely to have the highest risk within patients with CHD^6^ as, at least in complex cases, premature ageing of the heart may be present together with limited cardiac reserve, thus lacking the advantage of children that are, in general, less susceptible to symptomatic SARS-CoV-2 infections.

Heart transplantation/immunosuppression: while immunosuppression, in general, enhances the risk for viral infections and increased severity, as has been described for younger children with influenza, there’s no supporting evidence in SARS-CoV-2. Data from solid organ transplantation do not show that immunosuppression during the COVID-19 pandemic leads to a less favourable outcome. Immunocompromised children, for example, those treated for cancer, only rarely needed modifications of their treatment. Accepting that not all patients are tested for COVID-19 at least symptomatic worsening has not been observed.^34^ Nevertheless, transmission precautions are of paramount importance and should be strictly followed until further knowledge of SARS-CoV-2 impact on the immune system is available.^35^

## What is known about possible fetal COVID-19 transmission? Impact of postnatal testing and treatment especially in the setting of critical CHD?

Currently, criteria for vertical transmission have not been developed. Based on limited data, viremia rates in patients with COVID-19 appear to be low and transient, suggesting placental seeding and vertical transmission are unlikely.^36^ In a few recent review articles on pregnant women with COVID-19, no cases of vertical transmission have been registered.^37–39^ A few newborn COVID-19 cases have been reported based on elevated immunoglobulin M levels and/or pneumonia on days 1 or 2 of life, with either negative or not performed COVID-19 testing on fetal blood, amniotic fluid, and placenta.^40–43^ However, it is not reliable to diagnose congenital COVID-19 based only on immunoglobulin M detection because immunoglobulin M assays can be false-positive and cross-reactivity may occur. The most likely cause of positive immunoglobulin M in many of those cases could be related to early infant infection due to postnatal contact with infected parents rather than fetal transmission.^44^

Even though there is no reliable data to confirm vertical COVID-19 transmission, the Centers for Disease Control and Prevention recommend that newborns born to mothers with known COVID-19 at the time of delivery should be considered to have suspected COVID-19 and should be tested and isolated from other healthy infants.^45^ It is equally important to ensure that a neonate with critical CHD born to a mother with COVID-19 is managed in a timely manner and that testing for COVID-19 does not prolong or delay transfer to a tertiary centre with a neonatal CHD ICU able to deliver high quality and timely management of critical CHD.

## Which groups of medication are thought to be potentially effective for COVID-19 treatment in paediatric age group?

There is a lack of data supporting the efficacy of antiviral agents for the treatment of COVID-19 in children, though there are recommendations available from multicentre initial guidance on the use of antiviral agents in children.^46,47^ Given the typically mild course of paediatric COVID-19, supportive care alone is suggested for the overwhelming majority of cases. Antiviral therapy generally should be reserved only for symptomatic children or those with underlying conditions with a high risk of disease progression.

### Antiviral drugs

Remdesivir inhibits ribonucleic acid-dependent ribonucleic acid polymerase and has activity against coronaviruses. According to a multicentre panel, Remdesivir is preferred to other antiviral agents due to its better tolerance and beneficial effects in adults with COVID-19. Hence, the same assumption is being made for children, even though data regarding the benefits of Remdesivir for children with COVID-19 are lacking. On 22 October, 2020, the United States of America Food and Drug administration has approved Remdesivir to treat COVID-19 for use in adults and children 12 years of age and older and weighing at least 40 kg requiring hospitalisation.

Lopinavir/Ritonavir is not recommended for children due to the absence of efficacy and unfavourable pharmacodynamics and negative clinical trial data.^48^

### Immune modulators for adjunctive therapy

The benefits and risks of immune modulators (e.g., glucocorticoids, interleukin 6 inhibitors, interferon-beta 1b) and of convalescent plasma from recovered COVID-19 patients in the treatment of children with COVID-19 are uncertain. The use of these medications is referred only on a case-by-case basis according to disease severity. The routine use of COVID-19 patients is not recommended. The cytokine profiles of serum from some patients with moderate-to-severe COVID-19 overlap with those seen in macrophage activation syndrome and secondary hemophagocytic lymphohistiocytosis. Thus, interleukin 6 and interleukin 1 blockades and Janus kinase inhibition has been proposed as an approach to treat the systemic inflammation associated with severe COVID-19 illness.^49^ Corticosteroids are reserved only for critically ill patients with COVID-19. There is insufficient evidence for or against systemic corticosteroids for mechanically ventilated patients with acute respiratory distress syndrome (CI)*, and for adults with COVID-19 and refractory shock. The National Institutes of Health recommends using low-dose corticosteroid therapy (BII)*.^50^

The safety and effectiveness of dexamethasone for paediatric COVID-19 treatment have not been sufficiently evaluated as the Recovery trial did not include a significant number of children. According to the recent National Institutes of Health statement, dexamethasone is not generally recommended for children who require only low levels of oxygen support. On the other hand, dexamethasone may be beneficial in difficult cases where mechanical ventilation is required. Additional studies are needed to evaluate the use of steroids for the treatment of COVID-19 in children, including for multisystem inflammatory syndrome in children.^51,52^

### Anticoagulation

Patients with MIS-C are at risk of experiencing thrombotic complications. Especially in the setting of severe LV dysfunction, there is a higher risk for apical LV thrombus. And those with Kawasaki disease who have large or giant CA aneurysms are at risk for myocardial infarction. Additionally, hypercoagulability is associated with COVID-19, thus patients may be at risk for venous thromboembolism. A minimum required treatment for Kawasaki disease includes low-dose aspirin. Systemic anticoagulation may be used for patients with moderate-to-severe LV dysfunction. VTE prophylaxis should be recommended for older children and adolescents hospitalised with moderate-to-severe MIS-C only with calculated low bleeding risk. There is evidence that low-molecular-weight heparins appear to be associated with better prognosis in patients with moderate-to-severe COVID-19-induced coagulopathies or elevated D-dimer levels. A weak recommendation of subcutaneous enoxaparin use in mild to high risk of VTE was recommended in COVID-19 PICU guidelines.^53^ Anticoagulation therapy should be coordinated with paediatric intensivists, haematologists, and paediatric interventional cardiologists.^54^

There is no available data on the use of vitamin A for COVID-19; however, it is recommended to be used as an adjunctive agent for COVID-19 patients due to its effect on decreased morbidity and mortality from measles-associated pneumonia.^55^

*Rating of Recommendations: A = Strong; B = Moderate; C = Optional.

Rating of Evidence: I = One or more randomised trials with clinical outcomes and/or validated laboratory endpoints; II = One or more well-designed, non-randomised trials or observational cohort studies; III = Expert opinion.

## Are there medications to be avoided in paediatric and adult CHD patients that might worsen the outcome of COVID-19?

The potential experimental therapies for COVID-19 have important cardiovascular side effects and toxicities. Data for these side effects are extrapolated from patients treated for autoimmune diseases (Chloroquine/Hydroxychloroquine, Rocilizumab), hepatitis (Ribavarin, Interferon alfa), or human immunodeficiency virus infection (Lopinavir/Ritonavir).^18^

Azithromycin, Hydroxychloroquine, and Lopinavir/Ritonavir can cause conduction disorders, especially QT prolongation. The combination of Hydroxychloroquine and Azithromycin is not recommended due to a synergistic effect increasing the risk of severe arrhythmia and deleterious impact on the cardiovascular system.^50^ The QT interval should be monitored closely, especially in the setting of long QT syndrome with COVID-19.^5,56^ Other adverse cardiac events related to these drugs are less common, but include the following: ventricular hypertrophy, hypokinesia, heart failure, pulmonary arterial hypertension, and valvular dysfunction. Irreversible myocardial damage is seen in 12.9%, death in 30.8%, however, cardiac function normalises in the majority of patients (44.9%) upon withdrawal of Chloroquine and Hydroxychloroquine.^18,57^ On 17 June, 2020, the World Health Organization announced to stop the use of Hydroxychloroquine for COVID-19 patients, which was based on data from Solidarity and the Recovery trials. Both showed that Hydroxychloroquine does not result in the reduction of mortality of hospitalised COVID-19 patients, when compared with standard care.^58^

Lopinavir/Ritonavir may interact with antiplatelets, oral anticoagulants, digoxin, statins, and many others as it is a potent liver enzyme (CYP3A4) inhibitor.^59^

Tocilizumab has been shown to influence lipid metabolism in rheumatoid arthritis patients. However recently, the ENTRACE clinical trial supported the cardiovascular safety of Tocilizumab in these patients.^60^ Interleukin 6 targeting has not been tested for secondary prevention in cardiovascular disease.

There is no evidence that angiotensin-converting enzyme inhibitors/angiotensin II receptor blockers/angiotensin receptor neprilysin inhibitors or low-dose acetylsalicylic acid worsen outcomes in patients with confirmed or suspected COVID-19 or increase susceptibility to COVID-19. COVID-19 is not an indication to stop angiotensin-converting enzyme inhibitors/Angiotensin II receptor blockers/angiotensin receptor neprilysin inhibitors or low-dose acetylsalicylic acid as stopping these medications may cause worsening of patient’s heart condition, unless symptomatic hypotension or shock, acute kidney injury, or hyperkalemia appears.^12,50^ This applies to children, adolescents, and adults.

According to the British Congenital Cardiac Association, the Canadian Cardiovascular Society, and other organisations, patients with heart failure and hypertension should preferentially choose acetaminophen over non-steroidal anti-inflammatory drugs for fever or pain to avoid decompensation of these cardiovascular conditions; however, there is yet no firm evidence.^12,56^ Fever-triggered malignant ventricular arrhythmia is the major concern in Brugada syndrome with COVID-19 infection, therefore, fever should be aggressively treated with paracetamol.^31^

In patients with catecholaminergic polymorphic ventricular tachycardia and COVID-19, beta-blockers and flecainide should be continued with monitoring of drug interactions with antiviral drugs.^5^

## Preventive measures for CHD-patients? Should CHD patients of any age group be kept in strict and extensive isolation? What is the progress of COVID-19 vaccine development?

The British Congenital Cardiac Association has suggested that adults and children with CHD, within the groups mentioned in the answer of question 1, should be considered more vulnerable to COVID-19 and other infections, and should therefore be particularly strict in following the social distancing and hygiene measures.^12^

Based on definitions of social distancing, this would exclude the attendance of such individuals at nurseries, school, college, or universities. Socialising with family, going to restaurants, and children’s parties should be avoided as well.

Children who do not fall into the mentioned groups should not be more likely to become infected with COVID-19 and there is no evidence of higher risk of complications, thus general rules of preventive measures should be applied.^12^

On 22 September, 2020 the World Health Organization made an announcement of approximately 38 COVID-19 vaccines in clinical trials and 149 in preclinical evaluation.^61^

## What limitations are paediatric cardiologists facing in terms of CHD diagnostics and treatment in the presence of symptomatic COVID-19?

Not only have non-invasive cardiological diagnostic procedures been limited to urgent and emergency situations, but also paediatric/congenital catheterisation laboratories have dramatically reduced case volumes by postponing elective procedures.^33^ Hence, availability of catheterisation is limited in the vast majority of centres. Cardiac centres in regions with greater COVID-19 prevalence are more likely to delay urgent, but not emergency case types. The decision regarding urgent cardiac surgery is expected to be based on a multidisciplinary team decision and if possible, surgery should be delayed until the patient’s symptoms have improved and/or testing has been repeatedly negative.^62,63^

## Summary

The data regarding the COVID-19 crisis is being updated constantly. However, there is a scarce number of publications addressing the management of children with cardiac pathology. Hence, AEPC officers have been frequently asked various questions related to COVID-19 and patients with CHD. This led to the publication of FAQs and expert answers based on the most recent available literature sources. The goal of this document is to frame a discussion on how to take care of children with cardiac pathology in the setting of COVID-19 during this unprecedented crisis. As the times are changing quickly and information regarding COVID-19 is very dynamic, continuous collection of evidence will help guide constructive decision-making.
